# Glycinebetaine Biosynthesis in Response to Osmotic Stress Depends on Jasmonate Signaling in Watermelon Suspension Cells

**DOI:** 10.3389/fpls.2018.01469

**Published:** 2018-10-12

**Authors:** Zijian Xu, Mengli Sun, Xuefei Jiang, Huapeng Sun, Xuanmin Dang, Hanqing Cong, Fei Qiao

**Affiliations:** ^1^Hainan Key Laboratory for Sustainable Utilization of Tropical Bioresources, Institute of Tropical Agriculture and Forestry, Hainan University, Haikou, China; ^2^Key Laboratory of Crop Gene Resources and Germplasm Enhancement in Southern China, Ministry of Agriculture, Danzhou, China; ^3^Tropical Crops Genetic Resources Institute, Chinese Academy of Tropical Agricultural Sciences, Danzhou, China

**Keywords:** *Citrullus lanatus*, glycinebetaine, osmotic stress, JA signal, gene expression, HPLC

## Abstract

Glycinebetaine is an important non-toxic osmoprotectant, which is accumulated in higher plants under various stresses. The biosynthesis of glycinebetaine achieved via is a two-step oxidation from choline and betaine aldehyde, catalyzed by choline monooxygenase (CMO) and betaine aldehyde dehydrogenase (BADH), respectively. Up-regulated gene expression of BADH and CMO induced by stress is clearly observed, but the signal transduction is poorly understood. Here, glycinebetaine accumulation in response to osmotic stress and growth recovery induced by exogenous glycinebetaine were observed in a watermelon cell line. When tracing back to the genome sequence of watermelon, it shows that there exists only one member of *ClCMO* or *ClBADH* corresponding to glycinebetaine biosynthesis. Both genes harbor a CGTCA-motif in their promoter region which is involved in methyl jasmonate (MeJA)-responsiveness. Amongst MeJA, Ethephon, abscisic acid (ABA), and salicylic acid (SA), MeJA was most effective in gene inducing the expression of *ClCMO* and *ClBADH*, and the accumulation of glycinebetaine could also reach an amount comparable to that after osmotic stress by mannitol. Moreover, when ibuprofen (IBU), a JA biosynthesis inhibitor, was pre-perfused into the cells before osmotic stress, glycinebetaine accumulation was suppressed significantly. Interestingly, newly grown cells can keep a high content of glycinebetaine when they are sub-cultured from osmotic stressed cells. This study suggests that osmotic stress induced glycinebetaine biosynthesis occurs via JA signal transduction and not only plays a key role in osmotic stress resistance but also contributes to osmotic stress hardening.

## Introduction

Plants are subject to various stress conditions in their life cycle. Osmotic stress is one of the most common adverse environments encountered by higher plants. It can, e.g., lead to damage of cellular structures, disorganization of membrane, transformation of cytoskeleton, reduction or inactivation of enzyme activity, accumulation of reactive oxygen species (ROS) (Komis et al., 2001; Krasensky and Jonak, 2012). Responses to osmotic stress include a set of biochemical and physiological adaptations on the cellular level, such as changes of cell membrane composition, production of plant hormones and compatible solutes to change osmotic potential (Beales, 2004), and regulation of stress-related gene expression (Boudsocq and Laurière, 2005; Valliyodan and Nguyen, 2006) to counteract these negative impacts.

**FIGURE 1:**
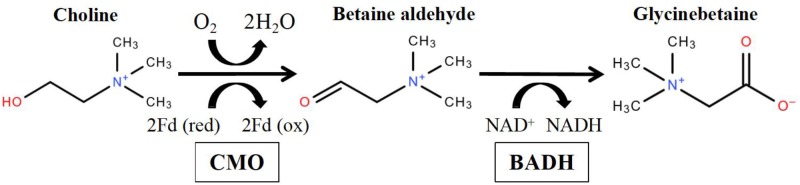
Biochemical pathway of glycinebetaine from choline via CMO (choline dehydrogenase) and BADH (betaine aldehyde dehydrogenase) in higher plants (Modified from Sakamoto and Murata, 2000).

Plants can accumulate glycinebetaine (GB, betaine), proline, trehalose and other compatible solutes to adjust osmotic homeostasis against salt stress, drought stress (McCue and Hanson, 1990) and even cold stress (Kishitani et al., 1994). Among them, one of the most extensively studied osmoprotectant is glycinebetaine, which is a soluble quaternary ammonium compound without cellular toxicity (McDonnell and Wyn Jones, 1988; Cleland et al., 2004). It has been suggested that glycinebetaine can stabilize membranes, protect proteins and photosystem II, and mitigate oxidative damage (Chen and Murata, 2011). Currently, glycinebetaine is widely found in bacteria, algae, higher plants and animals (Rhodes and Hanson, 1993; Prasad and Pardha-Saradhi, 2004). The biosynthesis of glycinebetaine can be divided by several pathways according to their precursors and enzymes (Sakamoto and Murata, 2000). In higher plants, glycinebetaine is synthesized as the result of a two-step oxidation of choline via the toxic intermediate betaine aldehyde (Cromwell and Rennie, 1953; **Figure 1**). The first step is catalyzed by choline monooxygenase (CMO), a Fe-dependent monooxygenase that contains Rieske-type [2Fe-2S] cluster-binding motif (Brouquisse et al., 1989; Burnet et al., 1995; Rathinasabapathi et al., 1997). It is also known as the rate-limiting step in glycinebetaine biosynthesis (Bhuiyan et al., 2007). The second step is mediated by betaine aldehyde dehydrogenase (BADH), a NAD^+^-dependent dehydrogenase which appears to be functionally identical to aminoaldehyde dehydrogenase (Wood et al., 1996; Fitzgerald et al., 2009). In previous reports, the gene expression of CMO and BADH were induced by salt (McCue and Hanson, 1992), drought (Russell et al., 1998; Li et al., 2016), cold (Xing and Rajashekar, 2001), and heat stresses (Alia Hayashi et al., 1998; Mitsuya et al., 2011) in various organisms. In some glycinebetaine-accumulating plants, e.g., Chenopodiaceae (Pan et al., 1981; Hanson et al., 1985; Weigel et al., 1986) and Amaranthaceae (Blunden et al., 1999), the glycinebetaine can be induced under salt or drought stresses and accumulated in chloroplast. However, biosynthesis of glycinebetaine in monocotyledon can be in peroxisome (Nakamura et al., 1997) or chloroplast (Hanson and Scott, 1980; Rhodes and Hanson, 1993).

Under osmotic stress, changes of turgor may initiate the signal transduction. The following secondary signals such as second messengers, stress hormones, and ROS then activate downstream signals through their own cognate receptors (Xiong and Zhu, 2002). Among secondary signals, many stress hormones have been reported playing key roles in osmotic stress signaling, such as abscisic acid (ABA, Skriver and Mundy, 1990; Zhu, 2002) and jasmonates (Riemann et al., 2015).

The accumulation of ABA induced by osmotic stress is well-known, and exogenous application of ABA can cause similar damage as osmotic stress does (Fujita et al., 2011). ABA can induce the expression of genes that associate with stress response and tolerance (Zhu et al., 1997) and promote the accumulation of compounds such as proline (Chen and Kao, 1993). These ABA-induced genes often contain the ABRE element in their promoter regions, which is a major *cis*-acting regulatory elements (CREs) in ABA-dependent gene expression (Yamaguchi-Shinozaki and Shinozaki, 2005). Many studies suggested ethylene also plays a pivotal role under stress (Müller and Munné-Bosch, 2015). However, negative regulation has been found in response to drought stress in *Arabidopsis* and maize since their drought tolerance enhanced as the result of reduced ethylene sensitivity (Shi et al., 2015). Jasmonates (JAs), important endogenous signal molecules, widely participate in wounding, insect attack, drought, salt stress, low temperature and other stresses in plant (Creelman and Mullet, 1995; Kazan, 2015; Riemann et al., 2015). Moreover, exogenous JAs can also alter drought tolerance in plants (Alam et al., 2014; Dhakarey et al., 2017) and promote the biosynthesis of certain secondary metabolites to respond osmotic stress (Gao et al., 2004; Zhou and Memelink, 2016). The JAs induced-genes contain hormone responsive *cis*-element CGTCA-motif (MeJA-responsive element) (Rouster et al., 1997). Genes in biosynthesis of osmoprotectants harbor both JA and responsive elements can be modulated synergistically. However, how stress signaling activating glycinebetaine synthesis remains as enigma.

Many previous reports showed that exogenous application of glycinebetaine improves the growth and fertility under various stress conditions in plants (Ashraf and Foolad, 2007; Lou et al., 2015). For examples, exogenously applied glycinebetaine improved growth and net photosynthesis under salt stress in maize (Yang and Lu, 2005). In addition, exogenous glycinebetaine treated bean plants improved the recovery ability, enhanced CO_2_ absorption and chlorophyll fluorescence under water deficit (Xing and Rajashekar, 1999). It also can alleviate the salt-induced inhibition of shoot growth and maintain the ultra-structure in rice seedlings (Rahman et al., 2002). And the protection mechanism of glycinebetaine can contribute to amelioration of ion homeostasis (Lutts et al., 1999) and regulation of cell osmotic pressure under salt stress (Deshnium et al., 1997; Shen et al., 2002). Although exogenous glycinebetaine has a positive effect on improving abiotic stress tolerance, its effect varies from species to species, and even show negative effects since it can stimulate the growth of pathogenic fungi and aphid in some cases (Ashraf and Foolad, 2007).

Watermelon (*Citrullus lanatus*) originated in tropical Africa (Dane and Liu, 2007) and the existing wild germplasm habited in Kalahari Desert has exhibited extraordinary drought tolerance (Kawasaki et al., 2000). Whether glycinebetaine contributes to this tolerance and which signal pathway is recruited for triggering glycinebetaine biosynthesis is dissected in this study with suspension cultured watermelon cells. Besides of evaluating the alleviation effect of exogenous glycinebetaine against osmotic stress, the gene involved in glycinebetaine synthesis in watermelon was characterized by means of bioinformatics and qRT-PCR. Furthermore, exogenous signal molecules combined with inhibitors were used to screen the signal pathway of the osmotic stress induced glycinebetaine biosynthesis. These results were discussed in context of the crosstalk of endogenous signals, abiotic stress resistance hardening and plant osmoprotectants.

## Materials and Methods

### Cell Line

Suspension cells of *Citrullus lanatus* were generated from tender leaves and cultivated in liquid medium containing 4.43 g l^−1^ Murashige & Skoog (MS) Basal Medium, Vitamins (PhytoTechnology Laboratories^TM^, United States), 30 g l^−1^ sucrose (Sangon, Shanghai, China), 1 mg l^−1^ 6-Benzylaminopurine (6-BA) (Sigma, Shanghai, China), and 0.2 mg l^−1^ 1-Naphthaleneacetic acid (NAA) (Sigma, Shanghai, China), pH 5.85. The suspension cells were incubated at 25°C in darkness on an orbital shaker (Kuhner Shaker, ISF4-X, Switzerland) at 150 rpm. The cells were subcultivated every 7 days by inoculating 5 ml of stationary cells into 15 ml of fresh medium in 50 ml Erlenmeyer flasks.

### Measurement and Analysis of Cell Growth Responses to Osmotic Stress

Packed cell volume (PCV) is used as indicator of the cell growth in determining suspension culture’s response to osmotic stress (Liu et al., 2013). PCV was measured at day 7 after subculture. D-mannitol (0–300 mM) was dissolved in MS medium. Glycinebetaine was dissolved in distilled water at a stock concentration of 10 mM, and the working concentration was 500 μM.

To demonstrate the effect of osmotic stress, cells were treated with different concentrations of D-mannitol (0, 50, 100, 200, and 300 mM) added during subcultivation. To determine whether glycinebetaine can mitigate the inhibition of cell growth caused by osmotic stress, glycinebetaine (10 mM) was supplied to cultivation medium containing different concentrations of D-mannitol during subcultivation.

### Sequence Analyses of *BADH* Gene and *CMO* Gene and Establishment of Phylogenetic Tree

The gene sequences of *BADH* and *CMO* of watermelon were searched from Cucurbit Genomics Database^1^ (Guo et al., 2013). The sequence analysis of *ClBADH* and *ClCMO* genes and their encoded amino acids was performed by online software. Promoter region and *cis* acting element site of the gene sequence were predicted by online analysis software Plant CARE^2^ (Lescot et al., 2002). The physical & chemical properties of amino acid sequences were analyzed by PortParam^3^ (Gasteiger et al., 2003), conserved domain and protein functions were predicted by Conserved Domain Database (CDD) and InterPro^4^ (Hunter et al., 2009). In addition, conserved sequence patterns of identify genes were described by Weblogo^5^ (Crooks et al., 2004) online software.

Phylogenetic trees of *BADH* and *CMO* gene encoding amino acid sequences were established, respectively. The amino acid sequences of the target genes carried on Blastp at National Center for Biotechnology Information (NCBI). Neighbor-Joining Tree (N-J Tree) was established with MEGA 7.0 software (Kumar et al., 2016). Genetic distance was calculated by Poisson calibration method (Zuckerkandl and Pauling, 1965), and tested with BOOTSTRAP (Felsenstein, 1985).

### Quantification of Gene Expression

Five genes including two glycinebetaine biosynthesis genes (the *BADH*; *CMO*), a JA-biosynthesis-related gene (allene oxide cyclase, *AOC*), and two genes of ethylene biosynthesis (1-aminocyclopropane-1-carboxylic acid synthase, *ACS*; 1-aminocyclopropane-1-carboxylate oxidase, *ACO*) were selected to determine the expression of genes after induction by mannitol.

Aliquots (1.5 ml) of cell suspension collected at day 4 after subcultivation were treated with D-mannitol with final concentration of 100 mM for 0, 0.5, 1, 2, 4, and 8 h, sedimented by low-speed centrifugation (4000 rpm; 5 min). The supernatant was removed and quickly frozen in liquid nitrogen (Ismail et al., 2012). Same procedure was used for 100 μM ethephon and MeJA treatment.

Samples were ground with steel beads by Tissue Lyser (Jingxin, Shanghai, China). Total RNA was extracted using the AxyPrep Multisource Total RNA Miniprep Kit (AxyPrep^TM^, Hangzhou, China) and the concentration and purity of mRNA was measured by a NanoVue Plus^TM^ (GE, CT, United States). The mRNA was transcribed into cDNA using the PrimeScript^TM^ RT reagent Kit with gDNA Eraser (Perfect Real Time) (Takara Bio, Japan) following instructions of the manufacture.

The ethylene biosynthesis inhibitor, (S)-trans-2-Amino-4-(2-aminoethoxy)-3-butenoic acid hydrochloride (AVG) (Sigma-Aldrich, Shanghai, China) (Yu and Yang, 1979), was dissolved in distilled water at 2 mg ml^−1^ as a stock solution. Ibuprofen (Sigma-Aldrich, Shanghai, China), inhibitor of jasmonate biosynthesis (Sudha and Ravishankar, 2003), was dissolved in dimethyl sulfoxide (DMSO) to a final concentration of 50 mM as a stock and Fluridone (FLU), an inhibitor of ABA biosynthesis (Stewart and Voetberg, 1987), was dissolved in distilled water to stock solution of 50 mM. To test whether these hormone-synthesis inhibitors can inhibit the expression of above mentioned genes under osmotic stress, the cells were pretreated with 10 μM AVG, 50 μM ibuprofen or 50 μM FLU for 30 min, respectively, before 100 mM D-mannitol was added.

The quantification of gene expression was determined by quantitative real-time RT-PCR (RT-qPCR). Transcripts were amplified by PCR primers that were designed by Primer Premier 5 and listed in **Supplementary Table S1**. RT-qPCR was carried out using PikoReal 96 Real-Time PCR System (Thermo Fisher Scientific^TM^, MA, United States). To normalize gene expression and quantify gene expression relatively, the 18s rRNA gene (HQ025803.1) of watermelon was used as the reference gene. Amplification of real-time RT-PCR products was detected by using the SYBR^®^ *Premix Ex Taq*^TM^ II (Tli RNaseH Plus) (Takara Bio, Dalian, China) according to the manufacturer’s instructions. Real-time RT-PCR cycling protocols comprised thermal cycling and data collection with a pre-denaturation step at 95°C for 7 min, followed by 40 cycles at 95°C for 5 s and at 60°C for 30 s, one cycle at 60°C for 30 s. For each sample, reaction was carried out in triplicate to ensure the reproducibility of results.

### Quantification of Glycinebetaine by HPLC

Aliquots (30 ml) of cell suspension were collected at day 4 after subcultivation and then treated with 100 mM of D-mannitol for 24 h. As a control, cells were treated with a corresponding amount of MS medium for the same time. The cells were filtered by suction flask and placed at −80°C refrigerator to quick-freeze for half-hour. A lyophilizer (Labconco, FreeZone, United States) was used to dry cells. A powder sample of watermelon cells was accurately weighed (1.0 g) and extracted with 20 ml of 80% methanol. The mixture was then sonicated for 60 min at room temperature and the supernatant was filtered through a 0.22-μm disposable needle filter. The stock solution of betaine (Sigma-Aldrich, Shanghai, China, ≥98%) was prepared in ultrapure water (10 mg/ml) and standard solutions of betaine were prepared with mobile phase to give concentrations of 10, 8, 6, 4, 2, and 1 mg ml^−1^, respectively.

Analysis of glycinebetaine was carried out on a high-performance liquid chromatograph (HPLC, Agilent, 1260 infinity, United States) equipped with a DAD detector as described previously (Zamarreño et al., 1997; Kalsoom et al., 2013). The column used in this study was a 5 μm Eclipse XDB-C18 phase column (250 mm × 4.6 mm) (Agilent, Shanghai, China). The mobile phase consisted of a 10:90 (v/v) water and acetonitrile mixed with 0.2% phosphoric acid at a flow rate of 0.5 ml min^−1^, oven temperature 35°C, detection wavelength 195 nm, and injection volume 2 μl.

## Results

### Establishment of a Suspension Cell Line of *Citrullus lanatus*

**FIGURE 2:**
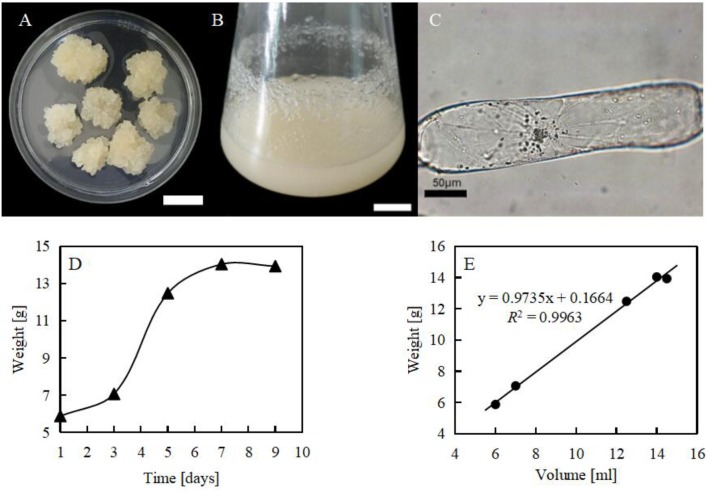
Suspension cell line of *Citrullus lanatus*. **(A)** Calli induced from tender leaves. Bar = 2 cm; **(B)** cell suspension culture of *Citrullus lanatus*. Bar = 2 cm; **(C)** suspension cultured cell, showing pronounced elongation of a single cell; **(D)** growth curve of cell lines; **(E)** volume and weight of suspension cultured cells.

With suspension cultured cells, cellular responses to both biotic and abiotic stress have been clearly dissected (Qiao et al., 2010; Ismail et al., 2012). In this study, the tender leaves of watermelon were selected to produce callus (**Figure 2A**). The soft callus was transferred to liquid medium to establish the suspension cell line (**Figure 2B**). From the microscopic observation, this suspension cell line was found to have a cylinder shape with elongated axis (**Figure 2C**). From the growth curve we could see that the subculture cycle of the suspension cells was 7 days, and the cells on the third and fourth day were in the exuberant exponential mitotic phase (**Figures 2D,E**).

### Exogenous Glycinebetaine Can Rescue Cell Growth Inhibition Caused by Osmotic Stress

**FIGURE 3:**
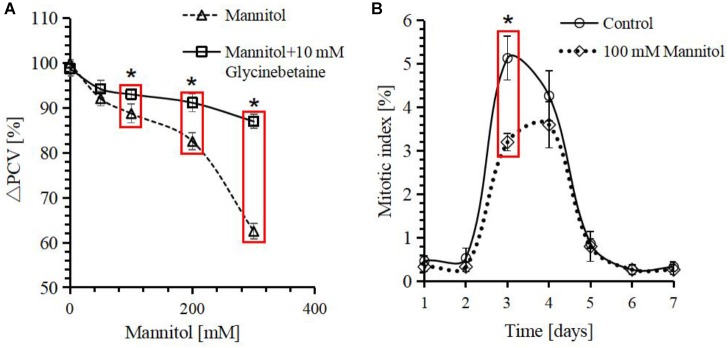
Response of *Citrullus lanatus* cell growth treated with mannitol indicated by relative packed cell volume (ΔPCV) and mitotic index. **(A)** Decrease in PCV caused by mannitol can be rescued by the addition of glycinebetaine. The PCV of the 0 mM mannitol is defined as 100%. **(B)** Cell mitotic indices over time after subcultivation. Each point represents the mean value from three independent experiments (*n* ≥ 500). Values represent averages from three biological measurements and error bars represent standard errors. ^∗^Indicates significant differences in values in red box by a one-side *t*-test at a confidence level of *p* < 0.05.

Glycinebetaine biosynthesis in response to osmotic stress has been widely observed in various organisms (Harinasut et al., 1996; Xing and Rajashekar, 1999; Giri, 2011). To dissect the protective mechanism of glycinebetaine against osmotic stress on cell growth, PCV was used as readout for cell growth inhibition when the cells were stressed by mannitol. When increasing the concentration of mannitol from 0 to 300 mM, the PCV decreased obviously (**Figure 3**). With 100 mM mannitol, the decrement was already over 10%, and with 300 mM mannitol, the decrement was close to 40%, when compared with cells cultivated under standard condition. However, when 10 mM exogenous glycinebetaine was added together with mannitol during subculture, the inhibition was alleviated significantly (**Figure 3A**), 300 mM mannitol could only cause about 10% decrement in presence of glycinebetaine.

Since PCV can decrease either as a result of inhibition of cell proliferation or as a result of cell expansion retardation, so the time course of mitotic index and PCV were plotted in presence or absence of 100 mM mannitol (**Figure 3B**). The PCV and mitotic index showed that osmotic stress inhibited both cell expansion and cell division.

### Sequence Analyses of *BADH* and *CMO* Gene

Betaine aldehyde dehydrogenase and CMO are the responsible for the first and second step in the glycinebetaine-biosynthesis pathway, respectively (**Figure 1**) and CMO is the only rate-limiting enzyme in the pathway (Sakamoto and Murata, 2000). By searching the Cucurbit Genomics Database, Cla019160 and Cla001775 were found to be the only members in *BADH* and *CMO* family, respectively.

The *ClBADH* gene is predicted to encode a 503-amino acid protein with molecular weight of about 54.5 kD and a pI value of 5.29. The protein classification is PNL02467 (ID:10010801) belonging to the ALDH-SF superfamily (**Supplementary Table S2**). The GO term prediction showed the biological processes were “metabolic process” (GO:0008152) and “oxidation-reduction process” (GO:0055114) and the molecular function was “oxidoreductase activity” (GO:0016491). The *ClCMO* gene is predicted to encode 417-amino acid protein with a molecular weight of about 47.2 kD and a pI value of 6.01. The protein classification was similar with choline monooxygenase (ID:10231827) and the superfamily prediction showed that it pertained to Rieske superfamily and SRPBCC superfamily (**Supplementary Table S3**). The GO term prediction showed the biological processes included “cellular aromatic compound metabolic process” (GO:0006725), “aromatic compound catabolic process” (GO:0019439) and “oxidation-reduction process” (GO:0055114). The molecular function was predicted as “iron ion binding” (GO:0005506) and “oxidoreductase activity” (GO:0016491). The biochemical properties of these two genes show highly similarity with the functional verified genes in sugar beet (McCue and Hanson, 1992; Russell et al., 1998), spinach (Weretilnyk and Hanson, 1989; Rathinasabapathi et al., 1997), sorghum (Wood et al., 1996), barley (Mitsuya et al., 2011), and other species (**Supplementary Table S4**).

To provide further information on gene regulatory networks, the promoter region was analyzed for CREs (Deokar and Tar’an, 2016). Besides containing the essential regulatory elements such as TATA-box, CAAT-box etc., the light responsive elements (Box 4, Box I, and GAG-motif), the plant hormone responsive *cis*-elements CGTCA-motif (involved in the MeJA-responsiveness) and the *cis*-elements responsive to biotic and abiotic stresses (HSE, MBS, and TC-rich repeats) were also found in both *ClBADH* and *ClCMO* (**Supplementary Tables S5, S6**). In addition, a *cis*-acting element ERE which is ethylene-responsive was identified in the promoter of *ClBADH* gene.

### Protein Sequence Alignment and Evolutionary Relationship Analysis

Through the phylogenetic relationship (**Supplementary Table S7** and **Supplementary Figure S1A**), we can see that the BADH protein of plants and *Escherichia coli* are distinguished from the phylogenetic tree and the protein sequence of *Citrullus lanatus* BADH is closer to the dicotyledonous plant. The alignments show that the BADH protein sequence of *Citrullus lanatus* is 93% homologous to that of *Cucumis melo* and *Cucumis sativus*, but only 41% homologous to that of *E. coli*.

Based on the available sequence of *Citrullus lanatus* BADH protein, the homologs of 12 species were identified by a Blastp search including *Beta vulgaris*, *Arabidopsis thaliana*, *Glycine max*, *Oryza sativa*, *Triticum aestivum*, *Zea mays* and others. Alignment of these proteins showed that they all contained a highly conserved decapeptide motif of aldehyde dehydrogenase, VTLELGGKSP, and cysteine residues (C) (**Supplementary Figures S1B,C**; Weretilnyk and Hanson, 1990; Ishitani et al., 1995). Previous studies showed that all monocotyledonous BADHs contain a C-terminal tripeptide SKL motif which is an indicator for peroxisomal localization (Reumann, 2004). Interestingly, they also contain an N-terminal QLFIDGE motif which is response for chloroplast localization (Weretilnyk and Hanson, 1990). However, activity of BADH was found higher in peroxisomes of monocotyledonous plant (Nakamura et al., 1997). *Citrullus lanatus* harbors both SKL and QLFIDGE motives, which indicates it may be localized in both chloroplasts and peroxisomes. Since cells in this experiment were cultured in dark, no chloroplast was observed, so, the BADH is more likely located in peroxisomes.

In the protein sequence of CMO, there are two conserved domains of the Rieske [2Fe-2S] domain and the Fe-binding motif (**Supplementary Figure S2B**). And the homology alignment results of CMO suggested that it is conserved compared to BADH of other plants. However, this step (choline→betaine aldehyde) is catalyzed by choline dehydrogenase (CDH) instead of CMO in *E. coli* (Landfald and Strøm, 1986) and mammals (Wilken et al., 1970; **Supplementary Table S8** and **Supplementary Figure S2A**). This conserved biosynthesis pathway in plants has been suggested as an evolutionary result of vertical descent from an angiosperm ancestor (Weretilnyk et al., 1989).

### Exogenous Signal Molecules Induced *ClBADH* and *ClCMO* Genes Expression and Glycinebetaine Biosynthesis

Regulatory elements in the promoter regions of *ClBADH* and *ClCMO* show that they respond to several signal transduction molecules including MeJA, ethylene, salicylic acid (SA), etc. (**Supplementary Tables S5, S6**). However, an ABA responsive *cis*-elements, which is widely involved in osmotic stress signaling, was not found in the promoter regions of *ClBADH* and *ClCMO*.

**FIGURE 4:**
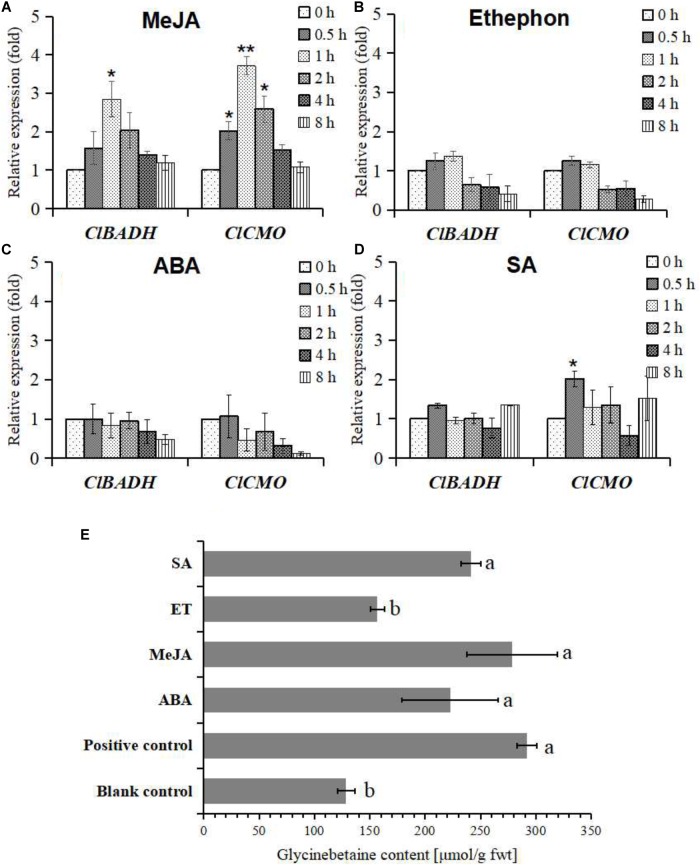
Differential expression patterns of *ClBADH* and *ClCMO* and changes of glycinebetaine content in watermelon cells subject to various hormone treatments. **(A)** A total of 100 μM MeJA treatment; **(B)** 100 μM ethephon treatment; **(C)** 100 μM ABA treatment; **(D)** 100 μM SA treatment; **(E)** the glycinebetaine contents were determined by HLPC with 100 mM mannitol, 100 μM ABA, 100 μM MeJA, 100 μM ethephon, and 100 μM SA for 24 h, respectively. The cells were treated with 100 mM mannitol as a positive control and equal volume MS medium as a blank control for 24 h. Data represent mean values and SEs from three biological measurements. ^∗^ and ^∗∗^ indicate statistical significant difference at confidence levels of *p* < 0.05 or *p* < 0.01 by a one-side *t*-test. Values with different superscripts are significantly different at each treatment (*p* < 0.05).

Here, exogenous MeJA, ethephon, ABA, and SA are used as signal molecules to induce the expression of glycinebetaine biosynthesis genes and to elicit the accumulation of glycinebetaine in watermelon cells. Compared with the solvent control, the expression of *ClBADH* and *ClCMO* genes were only up-regulated significantly in several time points after application of MeJA or SA (**Figures 4A–D**). The accumulation of glycinebetaine was also quantified by HPLC after induction by exogenous hormones (MeJA, ethephon, ABA, and SA) for 24 h. As shown in **Figure 4E**, MeJA, ABA, and SA all cause significant accumulation of glycinebetaine. However, when compared with the treatment of 100 mM mannitol, only MeJA treatment caused a comparable increase in glycinebetaine content, while other treatments could only induce weaker accumulation (**Figure 4E**). These results indicate that the glycinebetaine biosynthesis can be mainly intermediated by jasmonic acid signaling pathway.

### Osmotic Stress Can Enhance Jasmonic Acid Biosynthesis in Watermelon Cell

Since MeJA could induce equivalent amounts of glycinebetaine as osmotic stress, we tested whether the osmotic stress induced glycinebetaine biosynthesis was dependent on jasmonic acid related gene expression. The expression of two marker genes for glycinebetaine biosynthesis (*ClBADH* and *ClCMO*), one for JA biosynthesis (*ClAOC*), two for ethylene biosynthesis (*ClACO* and *ClACS*) were quantified after challenged by 100 mM mannitol.

**FIGURE 5:**
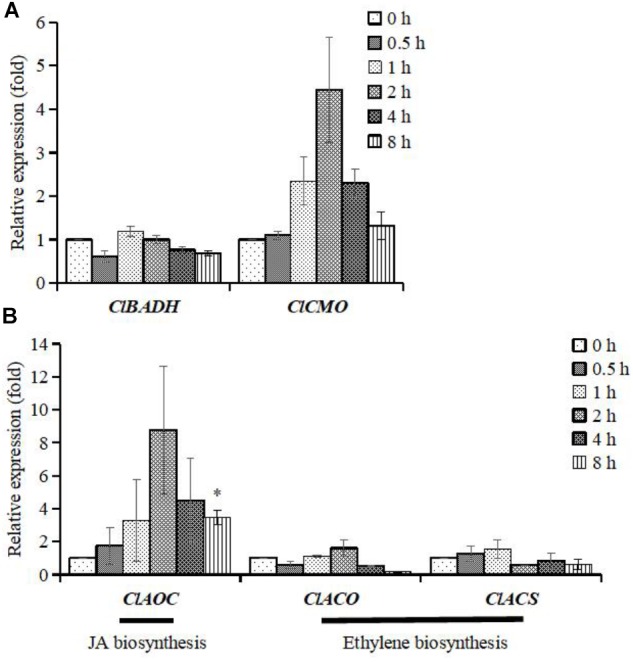
Differential expression patterns of genes at different time points in watermelon. The cells of watermelon were treated with 100 mM mannitol for 0.5, 1, 2, 4, and 8 h. **(A)** The expression patterns of *ClBADH* and *ClCMO*; **(B)** the expression patterns of *ClAOC*, *ClACO*, and *ClACS*. *ClAOC* is a marker gene for JA signaling and the *ClACO* and *ClACS* are two marker genes for ethylene signaling. Values represent average means from three independent measurements; error bars represent standard errors. ^∗^Indicates significantly difference (*p* < 0.05) between the data point and 0 h data point.

The *ClCMO* genes expression increased maximally ∼4-fold relative to the control in the experimental time frame of 0–8 h, whereas the *ClBADH* gene expression was almost not affected (**Figure 5A**). However, significant gene up-regulation of *ClAOC* was detected, which peaked at 2 h (∼9-fold relative to control). There were no obvious alterations of gene expression in the two marker genes for ethylene signaling (*ClACO* and *ClACS*) (**Figure 5B**), which is consistent with the fact that ethylene failed to accumulate glycinebetaine significantly (**Figure 4E**). Hence transcription of both, JA and glycinebetaine, biosynthesis genes are induced upon osmotic stress suggesting that JA might be involved in activation of glycinebetaine biosynthesis.

### JA Biosynthesis Inhibition Significantly Reduces Glycinebetaine Accumulation

**FIGURE 6:**
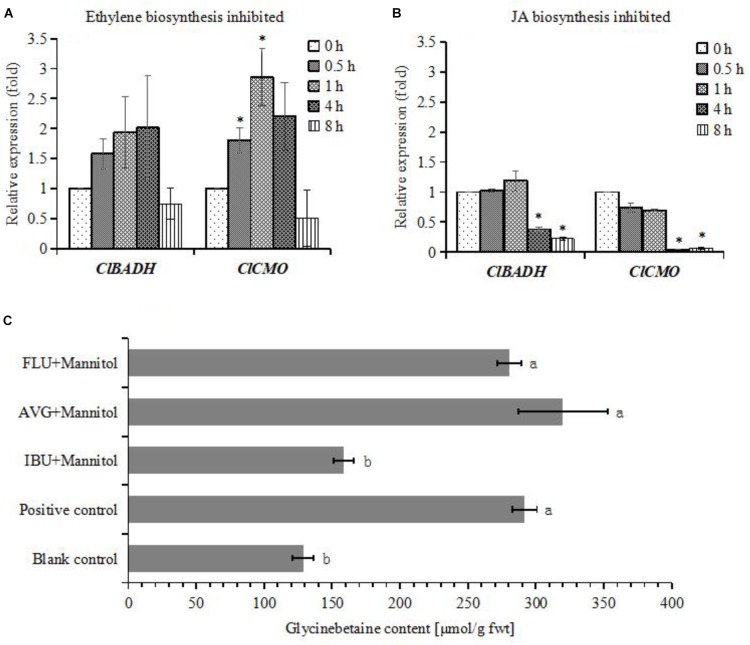
Differential expression patterns of *ClBADH* and *ClCMO* in watermelon cells under different treatments and glycinebetaine contents were determined. **(A)** The expression of *ClBADH* and *ClCMO*. The cells of watermelon were pretreated with 10 μM AVG for 30 min before adding 100 mM mannitol for 0.5, 1, 4, and 8 h. **(B)** The expression of *ClBADH* and *ClCMO*. The cells of watermelon were pretreated with 100 μM IBU for 30 min before adding 100 mM mannitol for 0.5, 1, 4, and 8 h. ^∗^Indicates significantly difference (*p* < 0.05) between the data point and 0 h data point. **(C)** The glycinebetaine contents were determined with HLPC. The cells of watermelon were, respectively, treated with 10 μM AVG, 100 μM IBU, and 50 μM for 6 h before adding 100 mM mannitol for 24 h. The cells were treated with 100 mM mannitol as a positive control and addition of equal volume MS medium as a blank control. Each values and SEs from three biological measurements. Values with different superscripts are significantly different at each treatment (*p* < 0.05).

To investigate whether JA biosynthesis play key role on glycinebetaine accumulation, cells were pretreated by ibuprofen (IBU), a JA biosynthesis inhibitor, just 6 h before adding mannitol. As the result, the glycinebetaine content was significantly decreased, and this phenomenon was not observed when cells were treated by AVG or FLU, which inhibit ethylene and ABA biosynthesis, respectively (**Figure 6C**). IBU also suppressed the expression of *ClBADH* and *ClCMO* (**Figure 6B**), but AVG enhanced the expression of these genes (**Figure 6A**). This strongly suggests that the osmotic stress induced glycinebetaine accumulation relies on newly synthesized JA, and ethylene may negatively regulate glycinebetaine biosynthesis.

### Glycinebetaine Accumulation and Drought Stress Hardening

**FIGURE 7:**
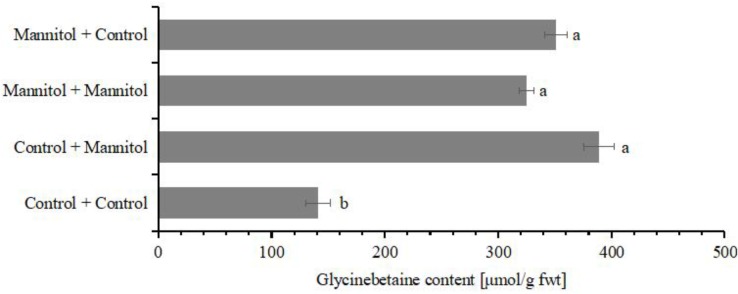
The accumulation of glycinebetaine with different treatment. The glycinebetaine contents were determined with HLPC. The cells of watermelon were treated with 100 mM for 24 h. The corresponding volume of MS medium was used as the control. Values with different superscripts are significantly different at each treatment (*p* < 0.05). All values are mean ± SE.

Drought hardening can greatly enhance the drought tolerance and increase the survival rate when plants inevitable encounter various abiotic stress in their life cycle (Yang S.L. et al., 2015). The increased tolerance contributes to various compatible osmolytes (Silva et al., 2010). Glycinebetaine has been found to be transported from old leaves to young leaves (Chen and Murata, 2011). In our suspension cultured cells, cells which were exposed to osmotic stress were subcultured into new medium with or without osmotic stress (**Figure 7**). Interestingly, the glycinebetaine content remained as high in both, cells subcultured in normal medium or such supplemented with mannitol (**Figure 7**). Thus, once exposed to osmotic stress the cells maintained their glycinebetaine levels high, probably as an adaptive mechanism.

## Discussion

Osmotic stress can cause a variety of stress responses in plants, including the production of osmolytes and the accumulation of phytohormone (Beales, 2004). Glycinebetaine was as one of the most intensively investigated compatible organic solutes in response to abiotic stress (Ashraf and Foolad, 2007). To respond to osmotic stress, plant hormones ABA, ET, and JAs were widely observed to induce the expression of stress related genes and accumulation of secondary metabolites (Zhou and Memelink, 2016). Here, with the newly established watermelon cell line, we found that osmotic stress caused growth inhibition could be rescued by addition of exogenous glycinebetaine, and osmotic stress caused accumulation of glycinebetaine is likely to be controlled by JA signaling.

The change of water potential can induce osmotic stress to plants in the environment, which harms cellular structures, or even lead to plant death (Xiong and Zhu, 2002). In this study, we found that mannitol-induced osmotic stress can inhibit cell growth by affecting mitotic index of suspension cultured watermelon cells during exponential division phase. However, the addition of exogenous glycinebetaine can ameliorate the inhibitory effect of osmotic stress on cell growth (**Figure 1**). Addition of exogenous glycinebetaine was confirmed to have a positive effect on plant growth and crop yield (Agboma et al., 1997), and glycinebetaine was also found to be translocated to all organs from exogenous application onto leaves (Mäkelä et al., 1996). However, exogenous glycinebetaine may increase the risk of pathogen attacks, as it is an effective growth substrate for plant pathogenic fungi (Ashraf and Foolad, 2007). For watermelon cells, osmotic stress retarded the cell growth and mitosis, whereas exogenous glycinebetaine can significantly recover it (**Figure 3**).

In higher plants, the *CMO* and *BADH* genes have been proved to be the key genes for glycinebetaine biosynthesis. By sequence homologous alignment, glycinebetaine biosynthesis has been identified in many plants (**Supplementary Figures S1, S2**). The CREs in these genes show that they can response to stress, including drought, salt, heat, and cold (Li et al., 2007; Zhang et al., 2008). As molecular switches, these elements are involved in the transcriptional regulation of various biological processes, including stress responses, and hormone responses (Yamaguchi-Shinozaki and Shinozaki, 2005). In watermelon, we observed that the glycinebetaine-biosynthesis genes contain not only the *cis*-elements responsive to stresses (HSE, MBS, and TC-rich repeats), but also some hormone responsive *cis*-elements, such as CGTCA-motif (involved in the MeJA-responsiveness) and ERE (ethylene-responsive element) (**Supplementary Tables S5, S6**). These various CREs suggest that glycinebetaine biosynthesis is involved in complex gene expression network and signal cascades.

The plant hormones can act as adaptive signals to induce gene expression under osmotic stress. In *Arabidopsis*, about 10% of the coding-genes are likely to be regulated by ABA (Nemhauser et al., 2006). Besides of these genes, many other genes also harbor ABA-responsive element, but they are controlled by an ABA-independent cascade (Shinozaki et al., 2003). In glycinebetaine biosynthesis, the expression of CMO and BADH has also been suggested as ABA-independent, respectively (Ishitani et al., 1995; Kalinina et al., 2012). In this study, we find that the promoter of *ClBADH* contains an LTR element (**Supplementary Table S5**), which is one of the major CREs in ABA-independent gene expression (Yamaguchi-Shinozaki and Shinozaki, 2005). Combining the fact that exogenous ABA cannot induce the gene expression of glycinebetaine biosynthesis in watermelon cells (**Figure 4C**), we conclude that the expression of *ClBADH* may be also ABA-independent.

The enhancement of the content of JAs has been observed after exposure to sorbitol simulated drought (Lehmann et al., 1995) and its bioactive conjugate JA-Ile can also be quantified in suspension cultured cells of *Vitis* after induced by elicitor (Chang et al., 2017). Here, MeJA-responsive elements (CGTCA-motif and TGACG-motif) were found in both the promoters of both *ClBADH* and *ClCMO*. Exogenous MeJA treatment can enhance the expression of these genes and lead to an increase in glycinebetaine content to a level even comparable as treated by mannitol (**Figures 4A,E**). Moreover, when the endogenous JA-synthesis was inhibited, both genes expression and glycinebetaine accumulation were inhibited (**Figures 6A,C**). Together they support that JA biosynthesis plays a dominant role in the activation of the *ClBADH* and *ClCMO* when challenged by osmotic stress and the influence of other hormones on glycinebetaine biosynthesis is indirect. However, exogenous appliance of SA and ABA also significantly enhanced the accumulation of glycinebetaine (**Figure 4E**), it is not consistent with the observation that the gene expression in glycinebetaine biosynthesis kept nearly stable (**Figures 4C,D**). Even the ABA biosynthesis was inhibited by FLU, the accumulation of glycinebetaine was increased significantly (**Figure 6C**). Since osmotic stress can affect the biosynthesis of plant hormones simultaneously (Takeuchi et al., 2011), the cross talk amongst ABA, SA, and JAs are still need to be further explicated.

Intriguingly, exogenous addition of ethylene can neither up-regulate the expression of glycinebetaine biosynthesis genes (**Figure 4B**) nor increase its content (**Figure 4E**). However, inhibition of ethylene-synthesis can slightly promote the accumulation of glycinebetaine (**Figure 6C**) and in the meantime enhance the expression of *ClCMO* (**Figure 6A**). In the complex defense signaling network, phytohormones JA, ethylene, and SA have been demonstrated with vital roles and their deliberative fine-tune determine the defense response (Yang Y.X. et al., 2015). The crosstalk between JA and ethylene can be synergistic or antagonistic (Zhu and Lee, 2015). Whether endogenous ethylene negatively regulates JA mediated biosynthesis of glycinebetaine will be clarified in our future research.

In addition, glycinebetaine biosynthesis in dicotyledons has been suggested to take place in chloroplasts, whereas monocotyledons possess biosynthesis enzymes in peroxisomes (Nakamura et al., 1997). Protein sequence alignment shows that the *ClBADH* contains a C-terminal sequence SKL, which is a typical signal peptide for localization to peroxisomes (Reumann, 2004). Since the cells were cultured under dark, and chloroplast is not formed or absent, therefore, the synthesis of glycinebetaine can occur in other plastids, such as peroxisomes (Nakamura et al., 1997). This suggests that even the non-green tissue also have the potential to synthesize glycinebetaine to improve drought tolerance.

Nowadays, more and more evidences indicate that plant have a memory mechanism which reflects the history of stimuli they were exposed to Ripoll et al. (2009). When plants encounter osmotic stress again, they can recall the stored information and which in turn play key roles in adaptation and acclimation. Unlike proline, an osmotic protectant, whose content decreases immediately after the stress is relieved; glycinebetaine remains stable in plants (Ashraf and Foolad, 2007). In this study, we showed that even the osmotic stress was removed; glycinebetaine is maintained to the same level irrespective of the osmotic potential in the solution (**Figure 7**). These data together support a new scenario on the role of glycinebetaine to drought-hardening, which is that plant not only can transport preexisting glycinebetaine to newly generated tissues (Mäkelä et al., 1996), but also can remember the previous stress stimuli and keeps glycinebetaine at high level in newly generated cells.

To sensing physical stimulus from abiotic stress is very different from sensing of chemical ligand (Wang and Nick, 2017), the physical stimulus must generate chemical signals to trigger on downstream signal transduction cascade. This process is proceeded in “susception” and perception, where physical stimulus transformed into an input passively and then this input is sensed and is capable to generate chemical signals (Nick, 2013). In plants, the distribution of this chemical signals is spatiotemporally heterogeneous. However, cell culture can provide a responsive model system to profile the early signaling events. With the newly established watermelon cell culture, we found that JA signaling play key roles in osmotic induced glycinebetaine biosynthesis. However, exogenous application of ABA also can effective induce cold hardness as glycinebetaine did in strawberry plants (Rajashekar et al., 1999). This indicates that the signal triggered on glycinebetaine biosynthesis can be varied in species. Moreover, in multicellular tissues or plants, this positive effect of exogenous applied glycinebetaine acts in a much more sophisticated way (Chen and Murata, 2011). In cold acclimation, signal molecular ABA can substitute low temperature to enhance cold tolerance and it is possible achieved by regulation genes associated with cold acclimation (Gusta et al., 2005). Glycinebetaine has been also suggested involved in manipulation gene expression (Chen and Murata, 2008). After application of 100 mM glycinebetaine on leaves and roots of *Arabidopsis*, enhanced gene expression was identified by DNA microarray analysis, which includes ROS-scavenging enzymes, transcription factors, membrane-trafficking components, and ferric reductase (Einset et al., 2007). Recently, glycinebetaine was also found playing great roles in enhancing salt tolerant by alleviate salt-induce potassium efflux (Wei et al., 2017). These accumulating evidences suggest the multiple roles of glycinebetaine in counteract the negative effects caused by abiotic stresses on plant growth. In the present study, exogeneous glycinebetaine showed promising effect on osmotic stress hardening in watermelon cells (**Figure 7**), we hypothesized that glycinebetaine may partially act as a signaling molecular in acclimation.

## Author Contributions

XJ and FQ designed the experiments. ZX, MS, HS, XD, and HC performed the experiments and analyzed the data. ZX and FQ wrote the paper. All authors read and approved the final manuscript.

## Conflict of Interest Statement

The authors declare that the research was conducted in the absence of any commercial or financial relationships that could be construed as a potential conflict of interest. The reviewer SC declared a shared affiliation, though no other collaboration, with several of the authors HS, XD, HC, FQ to the handling Editor.

## Footnotes

1 http://www.icugi.org/

2 http://bioinformatics.psb.ugent.be/webtools/plantcare/html/

3 http://web.expasy.org/protparam/

4 http://www.ebi.ac.uk/interpro/

5 http://weblogo.berkeley.edu/logo.cgi
