# Supplementary material for: Glycinebetaine Biosynthesis in Response to Osmotic Stress Depends on Jasmonate Signaling in Watermelon Suspension Cells
Source: Front Plant Sci. 2018 Oct 12;9:1469. doi: 10.3389/fpls.2018.01469 (PMC6194323; doi:10.3389/fpls.2018.01469)
Supplement: Supplementary file 1 [file Image_1.PDF]

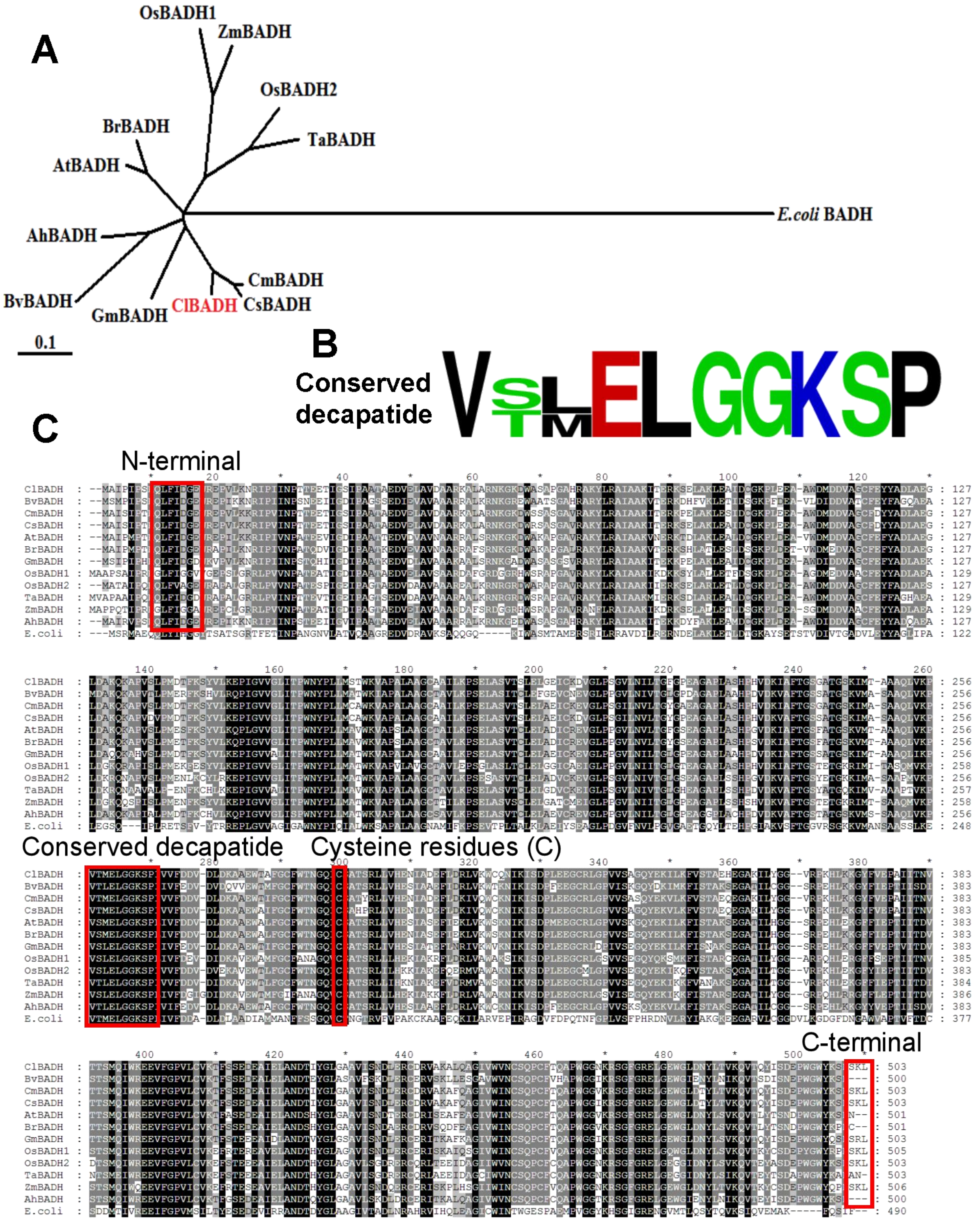

Figure S1 The bioinformatics analysis of BADH protein sequences from *C. lanatus*. (A) Phylogenetic tree based on Neighbor-Joining (NJ) and the BADH protein sequences (ClBAHD: Cla019160) of *C. lanatus* were searched from Cucurbit Genomics Database and the other BADHs were extracted from NCBI. Protein sequences were aligned with ClustalW and the phylogenetic tree was generated using bootstrap support based on 1000 replicates constructed using MEGA 7.0. (B) Consensus motifs of conserved decapatide domains. (C) Alignments of BADH protein sequences from *C. lanatus* and other species. The essential motifs, including N-terminal, conserved decapatide,Cysteine residues, and C-terminal sequences are indicated by *red frame*.

Abbreviation: CsBADH, *Cucumis melo*, BADH, NP 001284412.1; CmBADH, *Cucumis sativus*, BADH, AJF20760.1; AtBADH, *Arabidopsis thaliana*, BADH, NP565094.1; BrBADH, *Brassica rapa*, BADH, XP009104738.1; GmBADH, *Glycine max*, BADH, ADN03184.1; OsBADH1, *Oryza sativa* Indica Group, BADH, ABB83473.1; OsBADH2, *Oryza sativa* Japonica Group, BADH, ABI84118.1; TaBADH, *Triticum aestivum*, BADH, AAL05264.1; ZmBADH, *Zea mays*, BADH, ACG29220.1; AhBADH, *Amaranthus hypochondriacus*, BADH, AAB70010.1; *E.coli* BADH, *Escherichia coli*, BADH, AAA23506.1.
