# Supplementary material for: Glycinebetaine Biosynthesis in Response to Osmotic Stress Depends on Jasmonate Signaling in Watermelon Suspension Cells
Source: Front Plant Sci. 2018 Oct 12;9:1469. doi: 10.3389/fpls.2018.01469 (PMC6194323; doi:10.3389/fpls.2018.01469)
Supplement: Supplementary file 2 [file Image_2.pdf]

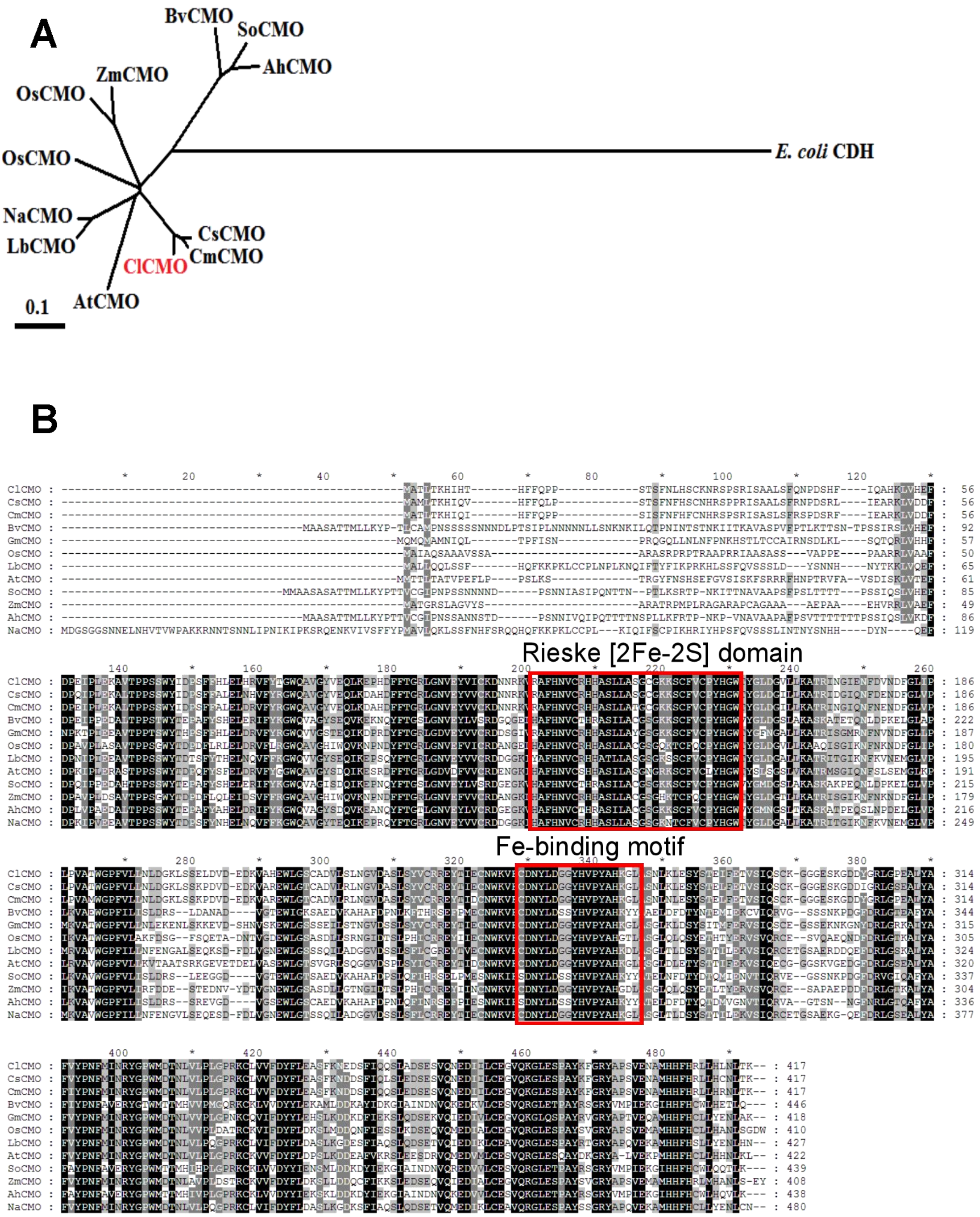

FIGURE S2 The bioinformatics analysis of CMO protein sequences from *C. lanatus*. (A) Phylogenetic tree based on Neighbor-Joining (NJ) and the CMO protein sequences (ClCMO: Cla001775) of *C. lanatus* were searched from Cucurbit Genomics Database and the other CMOs were extracted from NCBI. Protein sequences were aligned with ClustalW and the phylogenetic tree was generated using bootstrap support based on 1000 replicates constructed using MEGA 7.0. (B) Alignments of CMO protein sequences from *C. lanatus* and other species. The essential conserved motifs, including Rieske [2Fe-2S] domain and Fe-binding motif sequences are indicated by *red frame*.

Abbreviation: CsCMO, *Cucumis sativus*, ClCMO, XP004139149.1; CmCMO, *Cucumis melo*, CMO, XP008443631.1; BvCMO, *Beta vulgaris*, CMO, AAB80954.1; GmCMO, *Glycine max*, CMO,

XP003549280.1; OsCMO, *Oryza sativa* Japonica Group, CMO, CAE17671.1; LbCMO, *Lycium barbarum*, CMO, ACR15118.1; AtCMO, *Arabidopsis thaliana*, CMO, BAC21260.1; SoCMO, *Spinacia oleracea*, CMO, ABN43460.1; ZmCMO, *Zea mays*, CMO, NP001105926.1; AhCMO, *Atriplex hortensis*, CMO, AAF76895.1; NaCMO, *Nicotiana attenuata*, CMO, OIT26339.1 and *E.coli* CDH, *Escherichia coli*, CDH, APQ22920.1.
